# Assessment of municipal infrastructure development and its critical influencing factors in urban China: A FA and STIRPAT approach

**DOI:** 10.1371/journal.pone.0181917

**Published:** 2017-08-07

**Authors:** Yu Li, Ji Zheng, Fei Li, Xueting Jin, Chen Xu

**Affiliations:** 1 Institute of Geographic Sciences and Natural Resources Research, Chinese Academy of Sciences, Beijing, China; 2 University of Chinese Academy of Sciences, Beijing, China; University of Rijeka, CROATIA

## Abstract

Municipal infrastructure is a fundamental facility for the normal operation and development of an urban city and is of significance for the stable progress of sustainable urbanization around the world, especially in developing countries. Based on the municipal infrastructure data of the prefecture-level cities in China, municipal infrastructure development is assessed comprehensively using a FA (factor analysis) model, and then the stochastic model STIRPAT (stochastic impacts by regression on population, affluence and technology) is examined to investigate key factors that influence municipal infrastructure of cities in various stages of urbanization and economy. This study indicates that the municipal infrastructure development in urban China demonstrates typical characteristics of regional differentiation, in line with the economic development pattern. Municipal infrastructure development in cities is primarily influenced by income, industrialization and investment. For China and similar developing countries under transformation, national public investment remains the primary driving force of economy as well as the key influencing factor of municipal infrastructure. Contribution from urbanization and the relative consumption level, and the tertiary industry is still scanty, which is a crux issue for many developing countries under transformation. With economic growth and the transformation requirements, the influence of the conventional factors such as public investment and industrialization on municipal infrastructure development would be expected to decline, meanwhile, other factors like the consumption and tertiary industry driven model and the innovation society can become key contributors to municipal infrastructure sustainability.

## Introduction

Municipal infrastructure is a fundamental facility for the operation and development of an urban city and is of significant importance for improving the residence environment, city support capability and operation efficiency as well as for the stable progress of sustainable urbanization [1–4].

In recent years, research on municipal infrastructure has achieved substantial progress in the following areas: 1) assessment of transport infrastructure systems, including the cost-benefit of urban transport network [5, 6] and its environmental effect [7, 8], reliability [9] and management mechanism [6, 10, 11]; 2) study of urban water supply and drainage systems, including the influencing factors of the drainage system [10, 12] and its impact on urban river pollution [13, 14], and the reliability of water and electric power supply system [15, 16]; 3) study of urban energy infrastructure, including the requirements for urban energy infrastructure [17, 18] and its impact on the environment [19]; 4) urban environment and sanitation infrastructure, including the sustainability of municipal environment and sanitation infrastructure systems [20–23] and its impact on the environment [24–27]; and 5) study of ecological infrastructure in urban areas, including green area systems and green space in urban areas [1, 28–30]. On the scale of the whole country or an urban area, researchers have studied the influence of different factors, such as society, economy, climate, technology, on municipal infrastructure development and relevant administrative strategies [6, 11, 31, 32], and assessing municipal infrastructure systems and their sustainability has received increasing attention [1, 33]. Overall, much recent literature has focused on the evaluation of municipal infrastructure construction level, and tended predominantly to address assessing a limited range of infrastructure types, and often only one type [2, 4, 34], involving the factor analysis of risk assessment for one type of infrastructure [10], the efficiency assessment [35], ecological system service and life cycle assessment for the environment effects in an urban area [20, 21, 23, 36] and hierarchical analysis of infrastructure management [37]. Evaluation of a particular type of municipal infrastructure can only understand the special municipal infrastructure construction and its standard. And, municipal infrastructure development and the influences of various socio-economic factors are complex and systematic. Therefore, infrastructure assessment systematically is crucial to further understand the municipal infrastructure sustainable development, especially for China. Overall, comprehensive and multi-index urban infrastructure assessment on the scale of a region, or a country is relatively scarce, which can be particularly true for studies on municipal infrastructure construction in developing countries and emerging economies under transformation. Especially, few of these studies analyzed the question of accessing urban infrastructure integrally on such an emerging and transforming economy scale as China’s. Thus, the development of urban infrastructure to meet future livelihood demands is unclear and contested in many countries, including China. Assessing multiple-index municipal infrastructure development, focusing on its influencing factors based on samples from multiple cities needs further investigation critically in a large number of developing countries, including China.

After the Reform and Opening-up policy, urbanization in China has experienced rapid development. During 1980–2014, the proportion of the urban population in China grew from 19% to 54% [38, 39]. With the progress of urbanization, municipal infrastructure has attracted unprecedented attention in China. In 2013, the Guideline on Boosting Urban Infrastructure Construction by State Council was published, and municipal infrastructure construction had achieved rapid progress [2]. In 2015, urban infrastructure investment in China exceeded around CNY nine trillion [39]. However, compared to developed countries and regions such as Europe and America, China, as the world’s largest developing country and a typical emerging economy under transformation, still has a relatively low level of overall municipal infrastructure development, with a still rapid and special growth in the future. Understanding the determinants of China’s municipal infrastructure development is a key to directing future construction and investment for new projects. Meanwhile, China’s experience might also provide conceivable references and lessons for other similar countries where large-scale infrastructure investments and urbanization are being planned, especially such as some developing countries.

Generally, economic growth and its structural change, urban population, technology and consumption can significantly influence the municipal infrastructure construction as shown in the literature [2, 11, 20, 26, 31]. And it is very meaningful for further improving the municipal infrastructure to judge its determinants for cities at different urbanization and economic stages in China and other similar countries. Therefore, a systematic assessment of the municipal infrastructure sustainable development in China is firstly performed. Then, besides the above influencing factors, urbanization, industrialization, income and investment, especially national public investment, would also be especially critical to municipal infrastructure development in China, with a typical transformation feature [2, 22, 34, 37]. Based on the comprehensive assessment results from the factor analysis (FA) for municipal infrastructure in the prefecture-level cities (excluding principal capitals) in China in 2010, an optimized Stochastic Impacts by Regression on Population, Affluence and Technology (STIRPAT) model is employed to quantitatively examine the key driving forces for municipal infrastructure development at various stages of urbanization as firstly proposed by Northam [40] and economic development. The remaining sections of the article are structured as follows. First, the methodology of this study is presented, including the FA model, STIRPAT model and index system; then, China’s municipal infrastructure sustainable development is assessed and its influencing factors are explored; finally, conclusions are presented, and some policies are proposed and discussed.

## Methodology

### FA and STIRPAT

Since Escofier and Pagès [41, 42] applied a factor analysis (FA) model to multi-variable dimension reduction, the factor analysis method has been widely applied to the assessment of natural and human systems. The factor analysis model reassembles original variables to identify common variable influencing factors and to simplify data. Rotation is applied so that the factor variable becomes more intuitive and has a clearer meaning [43, 44]. First, this study constructs a FA (factor analysis) model to assess the overall development of municipal infrastructure. Let *X*_*i*_ (*i* = 1, 2, ···, *p*) be the original variable of municipal infrastructure development. Then, this variable is standardized to make the average 0 with a standard deviation of 1. *X* = (*X*_1_, *X*_2_, ···, *X*_p_)´ is an observable random vector of overall municipal infrastructure development. *F*_1_, *F*_2_, ···, *F*_m_ (*m*<*p*) are *m* factor variables. Components of vector *F* = (*F*_1_, *F*_2_, ···, *F*_m_)´ (*m*<*p*) are independent of each other, and the vector is unobservable. Vector *ε* = (*ε*_1_, *ε*_2_, ···, *ε*_m_)´ is independent of *F*, and *E*(*ε*) = 0, the covariance matrix of *ε* is a diagonal matrix. The components of *ε* are also independent of one another. Therefore:

$$\{\begin{array}{l} X_{1}=a_{11}F_{{}_{1}}+a_{12}F_{2}+\cdots +a_{1m}F_{m}+\varepsilon_{1}\\ X_{2}=a_{21}F_{1}+a_{22}F_{2}+\cdots +a_{2m}F_{m}+\varepsilon_{2}\\ \cdots \;\cdots \;\cdots \;\cdots \\ X_{p}=a_{p1}x_{1}+a_{p2}x_{{}_{2}}+\cdots +a_{pm}F_{m}+\varepsilon_{p} \end{array}$$ (1)

The above matrix is the constructed factor model for municipal infrastructure development. *F*_1_, *F*_2_, ···, *F*_m_ in the model are common factors (also called main factors) and *ε*_1_, *ε*_2_, ···, *ε*_m_ are unique factors of *X*_*i*_ (*i* = 1, 2, ···, *p*), which are components of vector *X*. Unique factors are independent of one another, and they are independent of all common factors. In the model, element a_*ij*_ in matrix *A* = (a_*ij*_) is the factor load; matrix *A* is the factor load matrix. The factor analysis model for comprehensive municipal infrastructure sustainable development is the assessment of load matrix *A* based on the sample data matrix in which the main component method is employed. The factor score is generated by the regression analysis based on least square method. The factor score can be calculated on the basis of linear combinations of the original scores of the surveyed objects weighted with the respective values from the factor score coefficient matrix.

The IPAT (Impact of Population, Affluence and Technology) proposed firstly by Ehrlich and Holdren [45] is a classic approach to assessing the impact of human activities. Therein, IPAT defines the main determinants of environment (*I*) as population (*P*), affluence (*A*, per capita production) and technology (*T*), or *I = PAT*, which has been employed in various studies, variations and model transformations in related works [46–49]. Although they are useful heuristic methods, these models are essentially accounting equations, and the test is not allowed because missing items are determined by known items, which is the most undesirable limitation [48, 50]. The relationship between anthropogenic factors and impacts should be verifiable via socio-ecological and empirical evidence rather than being simply assumed in the model [50]. To overcome this serious limitation, IPAT was reformulated into the stochastic model STIRPAT (Stochastic Impacts by Regression on Population, Affluence and Technology), as the pioneering work of Dietz and Rosa [46].

The STIRPAT model allows each coefficient to be estimated as a parameter, and each influencing factor can be decomposed. Based on the above formula, many studies made relevant improvements according to the respective research characteristics to conduct various studies on various cases. In this study, based on the characteristics of China’s municipal infrastructure and the relevant typical influencing factors, the STIRPAT model is constructed and employed to analyze the main influencing factors of municipal infrastructure in China. The non-proportional impact of variables can be statistically modeled as:

$$I\;=\;\alpha P^{\beta }A^{\gamma }T^{\delta }\theta$$ (2)

In the actual calculation, the logarithm is performed on both sides of the above formula as follows,

ln I=a+b ln P+c ln A+d ln T+∂ (3)

where ***a*** is a constant and ***∂*** is the error. ***I*** represents level of municipal infrastructure development; ***P*** represents urban population; and ***A*** stands for per capita GDP, fixed asset investment, per capita maintenance capital and others; and ***T*** represents industrialization level and industry structure level. Then, ***b***, ***c***, ***d*** represents the effects of various influencing factors, respectively.

### Data

Five thematic infrastructure areas are identified in the literature [51]: energy efficiency, waste management, sustainable urban transport, water/wastewater, and urban ecosystem management, and stressed that it’s important to integrate among sectors. Herein, based on five major urban infrastructure systems and ten municipal infrastructure development indexes (per capita road area, road network density, buses per 10,000 residents, drainage pipe density in built-up areas, water coverage, gas coverage, per capita gas consumption, green space ratio in built-up areas, green space coverage in built-up areas and water flush toilet ratio in built-up areas), factor analysis is performed for the municipal infrastructure sustainable development of the prefecture-level cities in China in 2010. Because many of the municipal infrastructure assessment indexes were only published by the national statistics service in 2011 but not in other years, this study is conducted based on data from 2010 to obtain a more comprehensive and objective result. However, until now, there are few changes for the whole China’s economy and urbanization model and the municipal infrastructure development model. So, the implications of empirical analysis and the conclusion still have a certain reference significance for policy debating.

The urbanization rate here represents the percentage of regional urban population to total population. The urbanization stages division of various cities are based on the three-stage urbanization theory proposed by Northam [40]. When urbanization rate is below 30%, it is at an initial stage of urbanization, in addition, the city is at an acceleration stage when urbanization rate is 30%-70%, and urbanization rate of above 70% means the city is at stable stage. The samples of the prefecture-level cities are divided into three groups: initial stage of urbanization (urbanization rate is below 30%), acceleration stage (urbanization rate is 30%-70%) and stable stage (urbanization rate is above 70%) as indicated Northam [40]. Because only a small number of cities in the sample have an urbanization rate over 70%, they are combined with cities in the acceleration stage of urbanization. This information can be combined with the index of per capita GDP in all the sampled cities to further divide the sample into some sample subgroups. The initial analysis shows that two of them, initial stage of urbanization with a low level of economic development, and acceleration stage of urbanization with a high level of economic development, are typical and representative sample groups. Therefore, this study primarily employs the STIRPAT model to analyze these two (Sample A and B).

All of the data are from the *China City Statistical Yearbook (2011)*, *China City Construction Statistical Yearbook (2010)* and *China Urbanization Survey Report (2011)*. The variables are listed in Table 1.

**Table 1:** Indexes and abbreviations.

Index	Abbreviation	Index	Abbreviation
**Per capita road area**	RPR	**Population**	P
**Road network density**	RD	**Per capita GDP**	A
**Buses per 10,000 residents**	B	**Urbanization rate**	UR
**Drainage pipe density in built-up areas**	DPD	**Industrialization**	IV
**Water coverage**	WPR	**Proportion of tertiary industry output in GDP**	SV
**Gas coverage**	GPR	**Per capita disposable income of urban residents**	IN
**Per capita gas consumption**	NP	**Total fixed asset investment**	FAI
**Green space ratio in built-up areas**	RGS	**Per capita city building and maintenance capital**	BM
**Green space coverage in built-up areas**	GR	**Water flush toilet ratio in built-up areas**	TR

## Results

### FA results

Before conducting the factor analysis of the data for China prefecture-level cities, the KMO test (Kaiser-Meyer-Olkin Measure of Sampling Adequacy) is first estimated. KMO is the index to compare the simple correlation coefficient and partial correlation coefficient, and it ranges from 0 to 1. When KMO is more close to 1, the correlation between variables is stronger, and the original variables are more suitable for factor analysis. This value is 0.643 at 1% significance level. Thus, the model is verified, and factor analysis can be performed. Then, the maximum variance orthogonal rotation method is applied. After rotation, the factors with an eigenvalue above 1 are selected. Four common factors are selected, and the total variance is over 65%. This essentially reflects the actual municipal infrastructure development. Each common factor’s corresponding eigenvalue and variance contribution rate are listed in Table 2. The load matrix after rotation is listed in Table 3.

**Table 2:** Eigenvalue and contribution rate of common factors for comprehensive municipal infrastructure development.

Common factor	Common factor load of original variable			Common factor load before rotation			Common factor load after rotation		
	Eigenvalue	Percentage of variance	Percentage of cumulative variance	Eigenvalue	Percentage of variance	Percentage of cumulative variance	Eigenvalue	Percentage of variance	Percentage of cumulative variance
**1 Eco-environment construction**	2.816	28.160	28.160	2.816	28.160	28.160	2.042	20.421	20.421
**2 Environmental sanitation**	1.394	13.940	42.100	1.394	13.940	42.100	1.680	16.801	37.222
**3 Road and transport**	1.231	12.312	54.412	1.231	12.312	54.412	1.628	16.279	53.500
**4 Residence infrastructure construction**	1.043	10.426	64.837	1.043	10.426	64.837	1.134	11.337	64.837

**Table 3:** Factor loading for comprehensive municipal infrastructure development before rotation.

Original assessment index	Main factor			
	*F*_*1*_	*F*_*2*_	*F*_*3*_	*F*_*4*_
**Green space ratio in built-up areas**	0.735	-0.606	-0.021	-0.132
**Green space coverage in built-up areas**	0.734	-0.577	-0.005	-0.210
**Gas coverage**	0.680	0.002	0.248	0.195
**Per capita road area**	0.519	0.448	0.320	-0.230
**Drainage pipe density in built-up areas**	0.572	0.424	0.190	-0.134
**Water flush toilet ratio in built-up areas**	0.255	0.366	0.350	-0.287
**Buses per 10,000 residents**	0.540	0.228	-0.466	0.455
**Road network density**	0.565	0.319	-0.496	0.239
**Per capita gas consumption**	-0.015	-0.120	0.426	0.590
**Water coverage**	-0.049	0.101	-0.514	-0.431

Thereafter, the factor structure is analyzed, i.e., the names and definitions of the common factors. According to Tables 3 and 4, the main component factors are “eco-environment factor,” “sanitation factor,” “road and transport factor” and “residence infrastructure construction factor.” The variance percentage of common factor represents the percentage of the common factor percentage in the total variance. And larger the variance percentage is, stronger the integrating capacity of the common factor.

**Table 4:** Factor loading for comprehensive municipal infrastructure development after rotation.

Original assessment index	Main factor			
	*F*_*1*_	*F*_*2*_	*F*_*3*_	*F*_*4*_
**Green space ratio in built-up areas**	0.954	0.041	0.121	0.013
**Green space coverage in built-up areas**	0.948	0.094	0.079	-0.040
**Gas coverage**	0.430	0.400	-0.310	-0.348
**Per capita road area**	0.092	0.776	0.115	0.007
**Drainage pipe density in built-up areas**	0.122	0.691	0.260	0.002
**Water flush toilet ratio in built-up areas**	-0.019	0.627	-0.098	-0.023
**Buses per 10,000 residents**	0.109	0.028	0.863	0.039
**Road network density**	0.103	0.155	0.817	-0.146
**Per capita gas consumption**	-0.033	-0.083	0.007	0.733
**Water coverage**	-0.031	-0.062	0.087	-0.671

Common factor *F*_1_ demonstrates a high degree of positive correlation with indexes such as green space ratio in built-up areas and green space coverage in built-up areas. *F*_1_ is defined as the eco-environment construction factor, whose variance percentage is 20.421%.

Common factor *F*_2_ demonstrates a high degree of positive correlation with indexes such as per capita road area, drainage pipe density in built-up areas and water flush toilet ratio in built-up areas. Because drainage pipes are normally constructed along roads, in the analysis, the index of per capita road area is included in common factor 2. Common factor 2 is defined as the environmental sanitation factor, whose variance percentage is 16.801%.

Common factor *F*_3_ demonstrates a high degree of positive correlation with indexes such as buses per 10,000 residents and road network density. *F*_3_ is defined as the road and transport factor, whose variance percentage is 16.279%.

Common factor *F*_*4*_ demonstrates a high degree of positive correlation with indexes such as per capita gas consumption and water coverage. *F*_*4*_ is defined as the residence infrastructure construction factor, whose variance percentage is 11.337%.

Furthermore, based on the selected common factors and percentage of common factor variance, different common factors are assigned with different weights, and the weighted sum is used to evaluate the municipal infrastructure development of prefecture-level cities (excluding provincial capitals) in China. The calculation formula is as follows:

$$EFI=\sum \omega_{i}\times v_{i}$$ (4)

In formula (1), *EFI* stands for the level of municipal infrastructure sustainable development; *ω*_*i*_ is the weight of common factor *i*, which is calculated from the proportion of each common factor’s variance percentage in the cumulative variance percentage; and *ν*_*i*_ is the score of factor *i* in the common factors.

**Fig 1:**
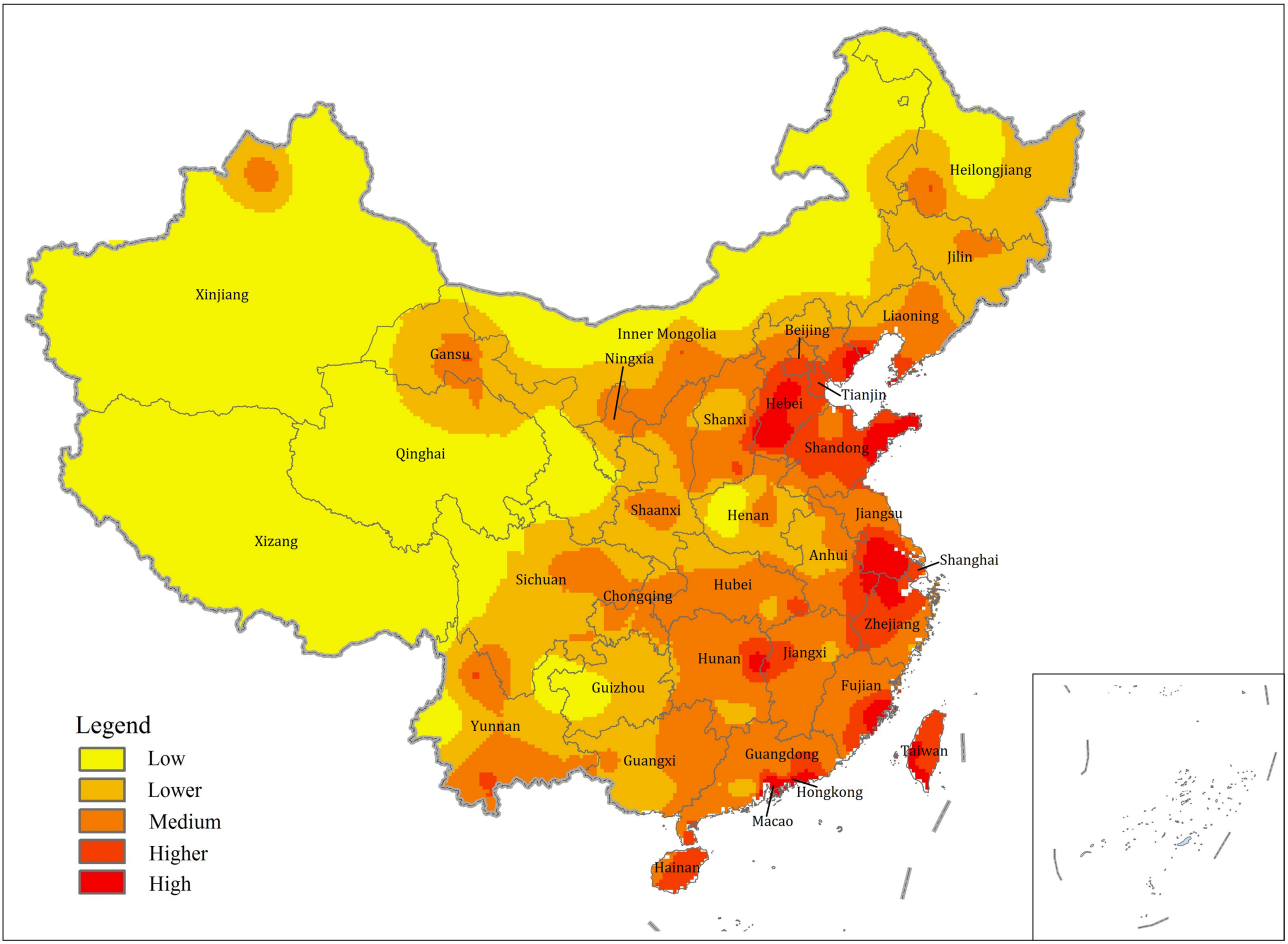
Distribution of municipal infrastructure development in China prefecture-level cities.

As is shown in Fig 1, municipal infrastructure development in China prefecture-level cities demonstrates typical characteristics of regional differentiation. That is normally in line with the distribution pattern of economic growth. Cities with high levels of overall sustainable development are primarily located in metropolitan areas including the Beijing-Tianjin-Hebei area, Yangtze River Delta, Pearl River Delta and Wuhan metropolis circle.

### STIRPAT results

In recent years, the relationship between municipal infrastructure development, economic growth and urbanization has attracted widespread attention [1, 2, 11, 26]. However, most researches primarily focused on the influence of a single factor of urbanization, economic growth on the municipal infrastructure, mainly in examples of developed countries with mature economies. Further studies could be needed to understand how various driving factors, such as population, industry and technology, influence municipal infrastructure sustainable development of cities at various stages of urbanization and economy, in developing countries, especially under economic transformation.

In this study, the STIRPAT model is employed to examine and analyze the influencing factors of municipal infrastructure development in various stages of urbanization and economic growth.

Firstly, we investigate the STIRPAT model for the total sample of the prefecture-level cities in China. The STIRPAT model is employed to investigate the influencing factors of the municipal infrastructure standard (*EFI*_*adj*_). The study primarily focuses on the main influencing variables such as urban population, per capita GDP, industrialization, proportion of tertiary industry output in GDP, per capita disposable income of urban residents, fixed asset investment, per capita city building and maintenance capital and urbanization level. In the STIRPAT model, *EFI* for the total sample is significantly influenced by BM, IN and FAI, with the significance of below 0.05. Herein, public investment is indicated to be still the main driving force of the municipal infrastructure. However, urban population percentage and tertiary industry cannot have significant effects, different from some developed countries.

Secondly, we mainly investigate the STIRPAT model for the sample group A in the initial stage of urbanization with a low level of economic development. As is indicated in the Table 5, *EFI* for cities in the initial stage of urbanization with a low level of economic development is primarily influenced by three major variables: IN, BM and IV, where the significances of all of the coefficients are below 0.01 and the model has a desirable fitting effect. Some variables, such as per capita GDP, per capita city building and maintenance capital and per capita disposable income of urban residents, have the positive influence on municipal infrastructure development of cities in the initial stage of urbanization with a low level of economic development. In addition, the coefficients show that industrialization contributes more than per capita disposable income of urban residents and per capita city building and maintenance capital, where elasticity for IN, BM and IV is 0.056, 0.086 and 0.334, mostly at a 1% level of significance, respectively, as listed in Table 5. Implicit here is that a 1% increase in IN, BM and IV, respectively, leads to a respective 0.056%, 0.086% and 0.334% increase in municipal infrastructure development in sample A cities of China.

**Table 5:** Results from the STIRPAT model for sample A.

	Non-standardized coefficient		Standard coefficient	T statistics
	B	Standard error		
**C**	-1.605** a	0.316		-3.650
**ln(IN)**	0.056***	0.011	0.453***	3.865
**ln(BM)**	0.086***	0.023	0.352***	2.980
**ln(IV)**	0.334***	0.068	0.460***	2.747

Note: ^a^ ***, ** and * denote significance below the 1%, 5% and 10% levels, respectively. UR, P, A, FAI, SV are not significant above 0.1.

Thirdly, we proceed to the STIRPAT model for the sample group B in the acceleration stage of urbanization with a high level of economic development. *EFI* for cities with a high level of urbanization and economy is primarily influenced by two variables: IN and BM. The significance of each coefficient is below 0.01 indicating a high level of significance. The municipal infrastructure development of cities in the acceleration stage of urbanization with a high level of economic development is significantly influenced by per capita disposable income of urban residents and per capita city building and maintenance capital in a positive way. In addition, the per capita disposable income of urban residents contributes more than per capita city building and maintenance capital, where elasticity for IN and BM is 0.232 and 0.048, at a 1% level of significance, respectively, as listed in Table 6. Implicit here is that a 1% increase in IN and BM, respectively, leads to a respective 0.232% and 0.048% increase in municipal infrastructure development in sample B cities of China.

**Table 6:** Results from the STIRPAT model for sample B.

	Non-standardized coefficient		Standard coefficient	T statistics
	B	Standard error		
**C**	-1.639	0.697		-2.350
**ln(IN)**	0.232*** a	0.077	0.374***	3.023
**ln(BM)**	0.048**	0.023	0.264**	2.132

Note: ^a^ ***, ** and * denote significance below the 1%, 5% and 10% levels, respectively. UR, P, A, IV, FAI, SV are not significant above 0.1.

## Conclusions and discussion

In the present study, based on the data of the prefecture-level cities in China, a FA analysis is performed to systematically assess municipal infrastructure sustainable development, and then a STIRPAT model is used to investigate the key influencing factors. The results indicate that municipal infrastructure in China has the typical characteristics of regional differentiation, in line with its economic development pattern, that is primarily influenced by income, industrialization and public investment. Municipal infrastructure development of cities in the initial stage of urbanization with a low level of economic development is significantly influenced by disposable income of urban residents, industrialization and city construction investment in a positive way, whereas in the acceleration stage of urbanization with a high level of economic development, it is significantly influenced by income of urban residents and city construction investment. China is a typical emerging economy and has displayed the phasic characteristics of a “catching-up economy”. The traditional growth model of investment and export, especially the former, are always the engines of China’s economic growth, since the economic take-off after the reform policy, and the city construction investment is critically influencing urbanization and the municipal infrastructure development, as the results of the empirical analysis.

In cities with a low level of urbanization and economy, municipal infrastructure development is significantly driven by disposable income of urban residents, proportion of secondary industry output in GDP and city building and maintenance capital in a positive way. When cities are in the initial and intermediate stages of urbanization, economic growth and industrialization are still the most significant influencing factors of municipal infrastructure. For primary development stage in developing and backward countries, industrial growth would critically influence municipal infrastructure development. Industrialization is a significant indicator on municipal infrastructure, especially applicable to that in the initial stages of urbanization with an economic boom, where secondary industry development in a city remains crucial. Moreover, even not in cities with a low level of urbanization and a high level of economy but also in cities with a high level of urbanization and economy, municipal infrastructure is still closely related to city construction investment and fixed asset investment. In China and other similar developing countries under transformation, public investment is still the primary driving force of national economic growth as well as the key influencing factor for urbanization. And, the income growth and public investment indicators remain the key constraint factors for municipal infrastructure sustainable development. However, urban population related consumption and tertiary industry still have limited effects in this country [4, 52]. And, the contribution of consumption to the municipal development is still so scant, while investment, especially national public investment, remains a key contributor to infrastructure sustainable development, which is the crux of the matter encountered by China and similar developing countries under transformation. On the whole, China’s urbanization relative consumption level, and the tertiary industry are less significantly related with the municipal infrastructure than some developed countries [4, 52]. Expectedly, the influence of the conventional national public service model and the investment driven and industrialization model on urbanization and municipal infrastructure development would continue to decrease, with secondary industry’s influence on municipal infrastructure development dropping off accordingly, while other relevant factors such as the consumption and tertiary industry driven model and the innovation society would be key to municipal infrastructure sustainable development, although for transforming countries it exists an obstacle to transform dominated public investment to consumption and private investment, with deep-rooted structural imbalance of difficult adjustment. Government decentralization progressively and increasing non-government economy are clearly needed to move forward firmly.

This study has provided a general overview of municipal infrastructure development in urban China, which is among the most challenging in the world due to the scale of population and economic activity and its transformation involved. Although a further investigation is clearly needed before any unquestionable conclusion can be drawn, especially comparative and a more detailed study of municipal infrastructure based on more panel data of China and other developing countries, deriving the quantitative estimates of the sustainable development and its likely critical influencing factors is helpful to advance decision debate. China’s experiences can also provide values for municipal infrastructure development in other developing countries.

## Appendix

Fifty-two prefecture-level cities in the sample A are in the initial stage of urbanization with a low level of economic development: Hebei Province—Hengshui and Xingtai; Shanxi Province—Lvliang, Linfen, Yuncheng and Xinzhou; Inner Mongolia Autonomous Region—Ulanqab; Heilongjiang Province—Suihua; Anhui Province—Bengbu, Chuzhou, Chizhou, Chaohu, Xuancheng, Suzhou and Buyang; Jiangxi Province—Ganzhou, Shangrao, Ji’an, Fuzhou and Xuanchun; Fujian Province—Putian; Shangdong Province—Heze; Henan Province—Anyang, Xinxiang, Jiaozuo, Luohe, Zhumadian, Nanyang, Zhoukou, Xinyang and Shangqiu; Hubei Province—Huanggang, Xianning, Xiaogan and Jingmen; Hunan Province—Shaoyang; Guangdong Province—Meizhou; Guangxi Zhuang Autonomous Region—Hechi, Yulin and Qinzhou; Sichuan Province—Luzhou, Ya’an, Neijiang, Nanchong and Suining; Yunnan Province—Zhaotong and Baoshan; Guizhou Province—Zunyi; Shaanxi Province—Hanzhong; Gansu Province—Pingliang and Tianshui; and Ningxia Hui Autonomous Region—Guyuan.

Sixty-one prefecture-level cities in the sample B are in the acceleration stage of urbanization with a high level of economic development: Hebei Province—Tangshan, Qinhuangdao, Langfang and Zhangjiakou; Shanxi Province—Yangquan; Inner Mongolia Autonomous Region—Ordos, Baotou, Wuhai and Tongliao; Liaoning Province—Dalian, Panjin, Anshan, Benxi, Yingkou, Fushun and Jinzhou; Jilin Province—Jilin and Tonghua; Heilongjiang Province—Daqing and Qitaihe; Jiangsu Province—Suzhou, Wuxi, Changzhou, Zhenjiang, Nantong, Lianyungang and Yancheng; Zhejiang Province—Ningbo, Shaoxing, Jiaxing, Zhoushan and Huzhou; Anhui Province—Ma’anshan, Tongling and Wuhu; Fujian Province—Xiamen and Sanming; Shangdong Province—Dongying, Qingdao, Yantai, Zibo, Weihai, Rizhao, Dezhou, Jining, Binzhou, Weifang and Tai’an; Hubei Province—Yichang; Guangdong Province—Dongguan, Foshan and Zhanjiang; Guangxi Zhuang Autonomous Region—Liuzhou; Hainan Province—Sanya; Shaanxi Province—Xianyang and Baoji; Gansu Province—Jiayuguan; Jiangxi Province—Xinyu and Pingxiang; and Ningxia Hui Autonomous Region—Shizuishan.
