# Correction: Assessment of municipal infrastructure development and its critical influencing factors in urban China: A FA and STIRPAT approach

**DOI:** 10.1371/journal.pone.0189530

**Published:** 2017-12-06

**Authors:** Yu Li, Ji Zheng, Fei Li, Xueting Jin, Chen Xu

There are errors in the Funding section. The correct funding information is as follows: This study was supported by the Science & Technology Basic Resources Investigation Program of China (2017FY101300), the National Natural Science Foundation of China (41271186, 41301642), the Foundation of Ethnic Group Culture Research Base of Nanling Corridor, Key Research Institute of Humanities and Social Sciences in Guangxi General Universities (2015KF03), and the Key Program of Chinese Academy of Sciences (ZDRW-ZS-2016-6-5). The funders had no role in study design, data collection and analysis, decision to publish, or preparation of the manuscript.
